# Supplementary material for: Genome of Alaskapox Virus, a Novel Orthopoxvirus Isolated from Alaska
Source: Viruses. 2019 Aug 1;11(8):708. doi: 10.3390/v11080708 (PMC6723315; doi:10.3390/v11080708)
Supplement: Supplementary file 1 [file viruses-11-00708-s001.zip › Supplement/viruses-548204-supplementary-legend_CG.docx]

**Figure S1.** Alignment of the left (A) and right (B) termini of the Alaskapox virus isolate genome with genomic termini of CPXV Brighton Red. Location of predicted CDS are shown by blue boxes. Position in the alignment is indicated above each graph. Percent identity of aligned sequences is shown by gray bars above each alignment. Aligned sequence is shown by black bars; gaps are shown by horizontal lines.

**Figure S2.** Comparison of AKPV011, 012, and 013 with reference OPXV sequences. Alignment of genomic region from AKPV011 to 013 with reference Old and New World OPXVs. Nucleotide identity to AKPV is shown by shading: identities are shown in gray, differences in black. Gaps not shared with AKPV are shown by horizontal black lines. Blue arrows indicate the locations of annotated coding sequences. Alignment position is shown above the graph.

**Figure S3.** Comparison of predicted A-type inclusion (ATI) protein from AKPV and reference OPXVs. Amino acid alignment is shown where sequences are compared to AKPV ATI protein: identities are shown in gray; differences are in black. Gray or black bars indication sequences, gray or black horizontal lines indicate gaps. Long black bars in reference sequences between position 700 and 900 represent sequence that is not present in AKPV (shown by gaps in AKPV). ATI sequences from OPVA, CPXV-B, CPXV-E1, AKMV-2013, ECTV-Moscow, SKPV, and RCNV-Herman are shown.

**Figure S4.** Potential recombination between Ectromelia virus (ECTV) and the Alaskapox virus isolate. Graph displaying the output of RDP4 bootscan analysis of Alaskapox virus, Orthopoxvirus Abatino (OPVA) and Raccoon poxvirus 85A (RCNV) with Ectromelia virus Moscow strain as the potential recombinant. Across the putative recombination region, AKPV displayed high support in two regions that can be seen as the pink AKPV line crossing the blue OPVA line at the putative recombination breakpoints.

**Figure S5.** Phylogenetic analysis of two suspect recombination regions and a neighboring control region. A. Phylogenetic tree based on 7,807 bp alignment preceding the first recombination region, as shown in Figure 4 and Figure S4. B – C. Phylogenetic trees based on two putative recombinant regions identified by RDP4 analysis, as shown in Figure 4 and Figure S4. Phylogenetic trees were estimated by Maximum Likelihood. Bootstrap values shown at nodes are based on 1000 replicates; scale is in substitutions per site.

**Table S1.** Reference genomes used in this study.

**Table S2.** AKPV annotation table. Each predicted coding sequence is compared to CPXV-BR and AKMV-2013; percent coverage and identity are based on blastn search.

**Table S3.** Blastp analysis to identify the most similar homolog to AKPV predicted proteins. The top blastp hit is shown for each AKPV predicted protein, including the species, strain, and accession number. Blastp was performed against the ‘non-redundant’ (nr) database.

**Table S4.** Sites under positive selection in AKPV187 (B5R homolog) as identified by codeml in PAML by Bayes Empirical Bayes analysis. The amino acid seen is reference sequences and in AKPV sequence is shown; position refers to the AKPV residue. Probability that omega (dN/dS) >1 (Prob(w>1)) is shown, where dN/dS>1 indicates positive selection.

**Table S5.** AKPV homologs of OPXV host range and virulence genes. Note, M13L family proteins are not found in OPXV genomes.
