# Supplementary figures and images for: Genome of Alaskapox Virus, a Novel Orthopoxvirus Isolated from Alaska
Source: Viruses. 2019 Aug 1;11(8):708. doi: 10.3390/v11080708 (PMC6723315; doi:10.3390/v11080708)

A

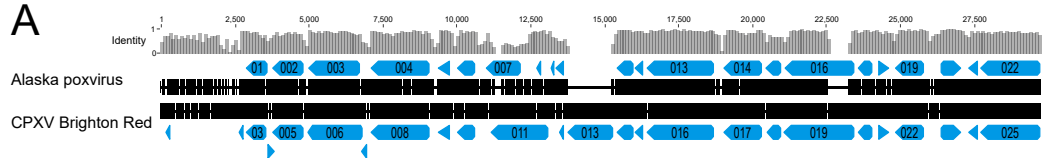

B

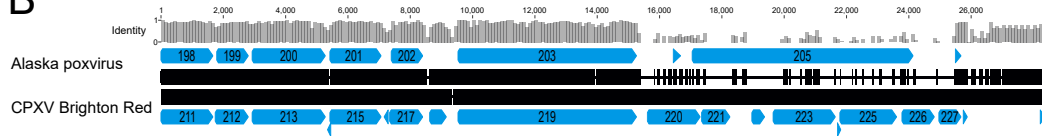

Supplement: Supplementary file 1 [file viruses-11-00708-s001.zip › Supplement/Figure S1.pdf]

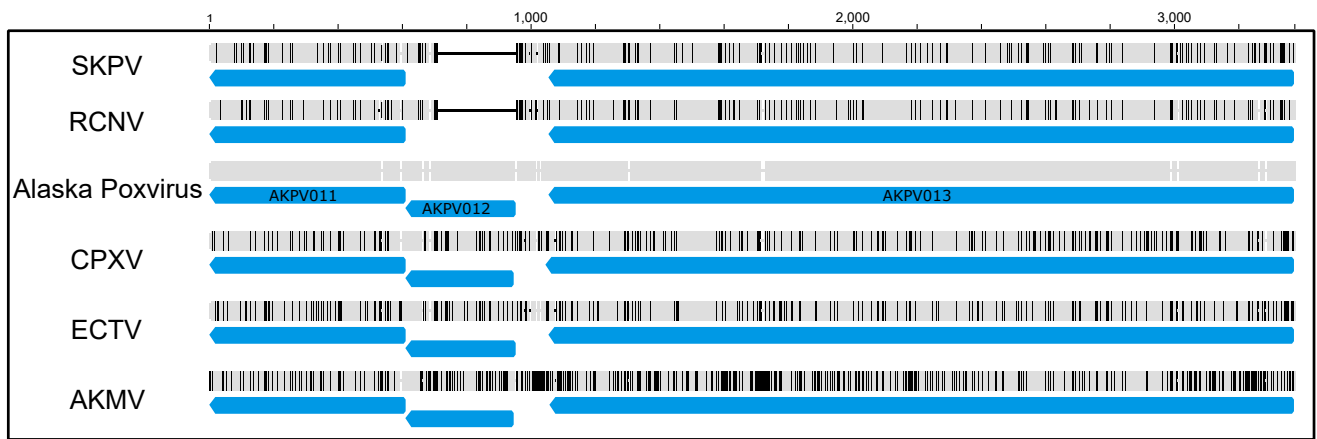

Supplement: Supplementary file 1 [file viruses-11-00708-s001.zip › Supplement/Figure S2.pdf]

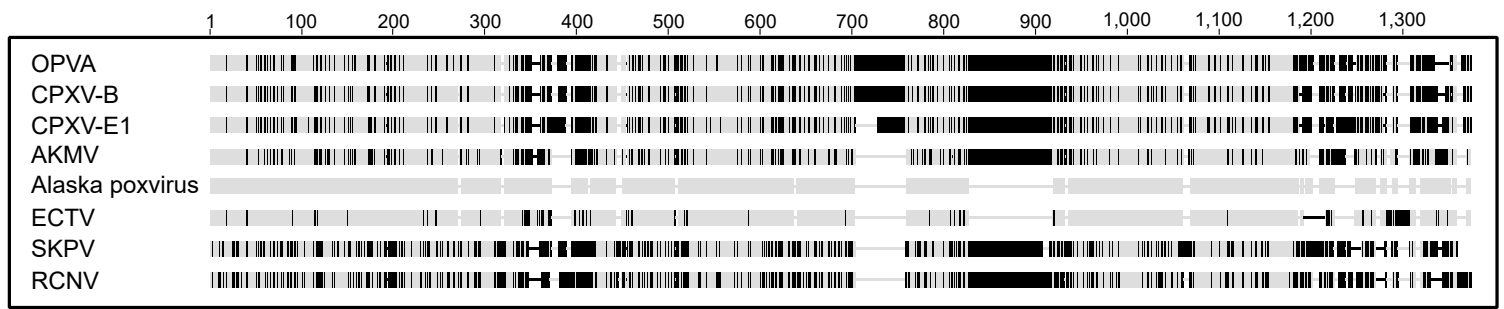

Supplement: Supplementary file 1 [file viruses-11-00708-s001.zip › Supplement/Figure S3.pdf]

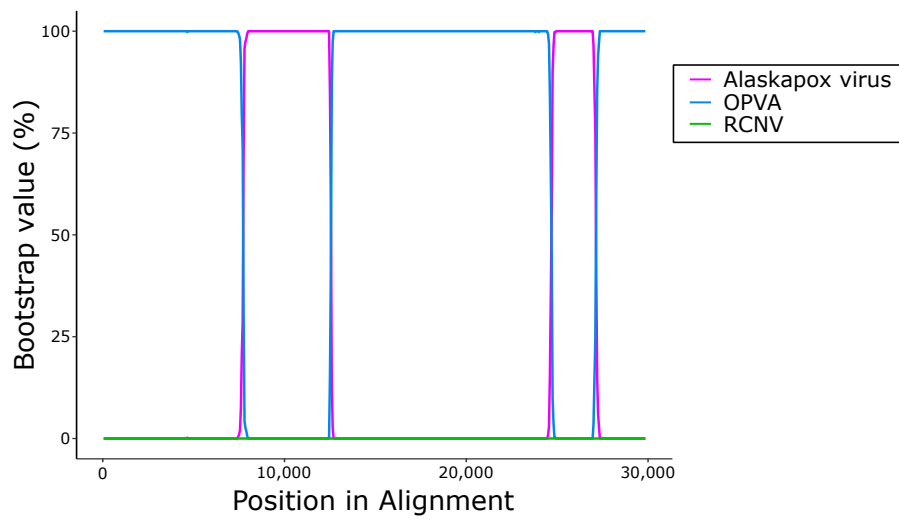

Supplement: Supplementary file 1 [file viruses-11-00708-s001.zip › Supplement/Figure S4.pdf]

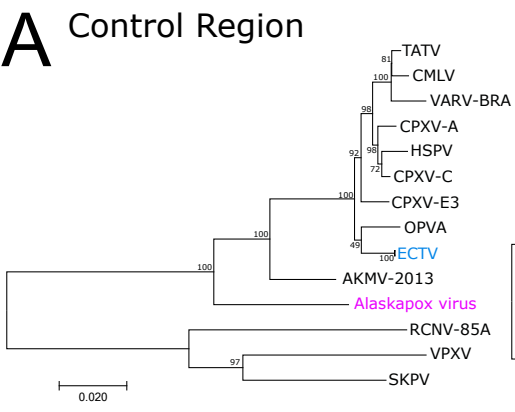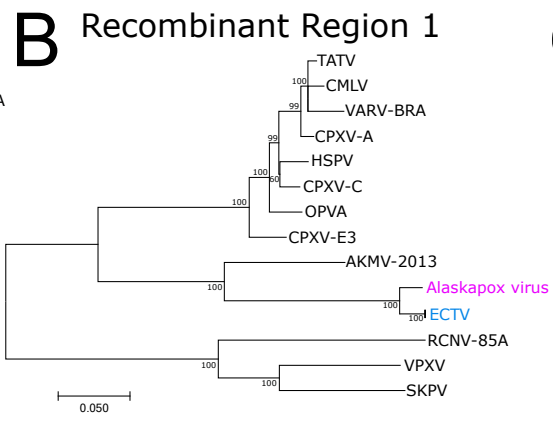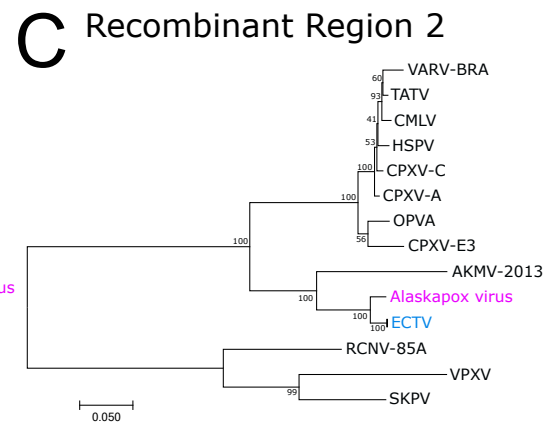

Supplement: Supplementary file 1 [file viruses-11-00708-s001.zip › Supplement/Figure S5.pdf]
